# Development and Validation of the Future Career Insecurity Scale (FCIS) in Law Students

**DOI:** 10.3390/bs15111590

**Published:** 2025-11-20

**Authors:** Cuiyu Lan, Xinying Weng, Qi-Lu Huang, Liqian Yu, Ruizhe Wang, Jie Su, Tianshu Zhou, Tingjian Lou, Yinlin Li, Wei Li

**Affiliations:** 1 Faculty of Law, Qingdao University, Qingdao 266071, China; lancyxie@qdu.edu.cn; 2 Faculty of Health and Life Sciences, University of Exeter, Exeter EX4 4PY, UK; xw456@exeter.ac.uk; 3 Department of Social and Behavioural Sciences, City University of Hong Kong, Hong Kong, China; qlhuang5-c@my.cityu.edu.hk; 4 Qingdao Medical College, Qingdao University, Qingdao 266071, China; yuliqian@qdu.edu.cn; 5 School of Social Science, Humanities & Law, Teesside University, Middlesbrough TS1 3BX, UK; e4445962@tees.ac.uk; 6 School of International Education, Guangdong Polytechnic Normal University, Guangzhou 510640, China; sujie@gpnu.edu.cn; 7 Faculty of Education, The University of Hong Kong, Hong Kong, China; u3629653@connect.hku.hk; 8 Department of History, School of Humanities, Tsinghua University, Beijing 100190, China; 12004001@zju.edu.cn; 9 Department of History, Hong Kong Shue Yan University, Hong Kong, China; 230046@hksyu.edu.hk; 10 Law School, Central University of Finance and Economics, Beijing 102206, China

**Keywords:** future career insecurity, law students, anxiety, self-doubt, uncertainty, scale validation, student mental health, screening

## Abstract

The transition from university to the workforce is a major developmental milestone that can generate significant psychological distress, especially for students in high-stakes professional programs such as law. Traditional measures of career-related anxiety often overlook the multidimensional nature of career insecurity and its culturally specific expressions. This study aimed to develop and validate the Future Career Insecurity Scale (FCIS), a novel instrument capturing three interrelated dimensions (future career anxiety, self-doubt, and uncertainty) among Chinese undergraduate law students. A two-study design was used with independent samples (*N* = 447 and *N* = 432). Study 1 applied exploratory factor analysis to identify the underlying structure of the FCIS. Study 2 conducted confirmatory factor analysis to validate the model and assess convergent validity using the measures of depression, anxiety, and stress. EFA supported a three-factor solution: Future Career Uncertainty, Self-Doubt, and Anxiety. CFA indicated good fit for a correlated three-factor model with satisfactory internal consistency (Cronbach’s *α* = 0.82–0.87). Convergent validity was evidenced by positive correlations between FCIS scores and DASS-21 depression, anxiety, and stress subscales. These findings indicate that the FCIS is a brief, multidimensional, and psychometrically robust measure of future-oriented career distress in legal education. Use of the FCIS can provide a brief, theory-aligned measure of future-oriented career distress in legal education and can support screening, targeted referral, and the evaluation of behaviorally informed interventions in university settings.

## 1. Introduction

Future career insecurity refers to an individual’s perceived vulnerability to adverse developments in their occupational future (Hamilton, 2021; C. Lee et al., 2018). In legal education, this insecurity is intensified by the intersection of high academic expectations, professional ambiguity, and a hypercompetitive job market (Krieger, 2002; Reed & Bornstein, 2013; Sheldon & Krieger, 2004). These pressures are often magnified in Confucian-influenced societies such as China, where success in prestigious professions is tightly coupled with family honor and social status (Strevens & Lai, 2023). The resulting strain is not simply a matter of occupational preference; it is a public health concern for universities because prolonged anticipatory stress is associated with internalizing symptoms and functional impairment in student populations.

The transition from university to practice is a particularly fragile period. Unlike fields with structured pipelines, the legal pathway features diffuse entry points and variable selection practices, which heighten perceived unpredictability and feed affective responses such as anxiety and self-doubt (Hamilton, 2021; Ng & Feldman, 2007; Savickas, 2020). Recent macro-level shifts, including post-pandemic market volatility and saturation in entry-level legal roles, further elevate concerns about employability and career sustainability (Ward, 2021; Zheng & Yan, 2024). These contextual pressures make law students an at-risk group for future-oriented distress that is likely to present in campus health and counseling services.

A growing literature links career insecurity with stress, depressive symptoms, sleep disturbance, and intentions to withdraw or change direction (T. J. Kim & von dem Knesebeck, 2016; Y. K. Kim et al., 2021; S. H. Lee & Jeong, 2017; Yaşlıoğlu et al., 2013). Law students may be especially vulnerable because formative identity work unfolds alongside normed ranking, competitive milestones, and rigid performance norms, which can reinforce fears of falling short despite objective achievement (Ferris, 2021). Understanding the specific dimensions of future career insecurity in this population is therefore essential for both theory and service provision (Chan et al., 2024; Wang et al., 2012; Yao & McWha-Hermann, 2025; Yuan, 2025).

### 1.1. Occupational Future Time Perspective as a Conceptual Lens

Socioemotional Selectivity Theory (SST) proposes that individuals’ goal orientations and emotional regulation shift depending on their perceived future time horizon: when time is perceived as limited, people tend to prioritize emotionally meaningful goals and become more present-focused, whereas an open-ended future supports exploratory, growth-oriented pursuits (Carstensen, 2006).

Building upon this foundation, the Occupational Future Time Perspective (OFTP) model extends SST to vocational contexts and offers a coherent framework for organizing these phenomena (Zacher & Frese, 2009). OFTP reflects the subjective sense of remaining time, opportunities, and constraints in one’s work life (Y. Chen et al., 2025a). A broad OFTP is associated with opportunity-seeking and adaptive self-regulation, whereas a constrained OFTP is linked to avoidance, heightened stress, and impaired goal pursuit (Gao et al., 2025).

In high-stakes academic settings such as legal education, compressed training timelines, intense gatekeeping, and uncertain job prospects can restrict students’ OFTP, increasing their vulnerability to cognitive and affective distress (Kooij et al., 2018). In this study, OFTP is used as the primary conceptual lens: we frame future career insecurity as a set of downstream cognitive–affective states that emerge when students perceive limited career time and opportunity. Our proposed tripartite framework aligns with the constraints and motivational focus dimensions of OFTP, offering a tailored structure for understanding psychological distress in career-preparatory educational environments.

### 1.2. Dimensions of Future Career Insecurity

Within the OFTP framework, future career insecurity can be conceptualized as a manifestation of constrained future time, reduced perceived opportunities, and heightened perceptions of constraint (Y. Chen et al., 2025a; Gao et al., 2025; Zacher & Frese, 2009). When students perceive that their occupational future is limited in scope or time, three interrelated cognitive–affective processes are activated. First, a narrowed perception of opportunities fosters future career uncertainty (FCU), as students experience ambiguity about available career pathways and outcomes. Second, perceived constraints on personal agency give rise to future career self-doubt (FCS), which is reflected in diminished beliefs about one’s competence and control over desired professional goals. Third, affective arousal stemming from temporal and opportunity constraints manifests as future career anxiety (FCA), a stress-oriented emotional reaction to anticipated failure or instability. These three facets together represent distinct yet theoretically coherent expressions of a constricted OFTP, translating its temporal-cognitive propositions into the lived experience of students in high-stakes professional training contexts.

#### 1.2.1. Future Career Uncertainty

FCU denotes apprehension about the predictability of legal career outcomes (Spurk et al., 2022). It is more likely when students perceive insufficient clarity or stability in professional pathways, for example, due to opaque hiring practices, oversupply of graduates, or fluctuating demand for legal services (Gao et al., 2025; Organ et al., 2016; Sheldon & Krieger, 2004). Cognitive Appraisal Theory posits that ambiguous, uncontrollable futures are inherently stressful, biasing attention and decision processes toward risk and away from exploration (Lazarus, 1984). Empirical studies associate higher uncertainty with disengagement, psychological distress, and difficulty committing to long-term goals (Y. Chen et al., 2024; Gao et al., 2025; Hong, 2025).

#### 1.2.2. Future Career Self-Doubt

FCS reflects diminished beliefs about one’s capacity to attain a desirable legal role (Kooij et al., 2018). Competitive evaluation, normed comparison, and perfectionistic standards can promote feelings of inadequacy even among high achievers (Jaffe et al., 2021; Skead & Rogers, 2014). The impostor phenomenon illustrates how self-doubt persists in elite contexts, as students attribute success to luck and fear exposure as a sign of inadequacy (Clance & Imes, 1978; Ochs, 2022). Within an OFTP lens, the co-occurrence of narrowed time and opportunity is likely to be associated with self-deprecating beliefs and withdrawal tendencies (Duru et al., 2023). These pressures may be intensified by strong familial expectations and culturally valued markers of prestige (Reed & Bornstein, 2013).

#### 1.2.3. Future Career Anxiety

FCA is an affective response to perceived instability, including fear of failure, worry about job acquisition, and dread of not meeting internal and social expectations (Y. Chen et al., 2024). In Confucian contexts, anxiety may be amplified by the salience of occupational prestige and filial obligations (Zhu et al., 2021). OFTP predicts that students who construe the future as constrained will exhibit greater anticipatory arousal and threat appraisal (Gao et al., 2025; Zacher & Frese, 2009). Intolerance of uncertainty, the dispositional tendency to experience ambiguous situations as aversive, can further exacerbate worry and avoidance, undermining planning, engagement, and well-being (Carleton et al., 2007; Y. Chen et al., 2024).

### 1.3. Limitations of Existing Measures

Despite increasing recognition of these concerns, validated measures that capture the career-specific cognitive and emotional profile of law students remain sparse. Instruments adapted from workforce samples often emphasize job status or general confidence, potentially overlooking the nuanced blend of uncertainty, self-doubt, and anxiety experienced by students whose trajectories are still forming and are closely shaped by institutional structures (Spurk et al., 2022). General mental-health scales detect severity but do not index future career cognitions; decision-making or self-efficacy instruments address related constructs but typically omit sustained anticipatory arousal. As a result, researchers and campus services lack a brief, multidimensional tool that aligns with theory, reflects lived experience in high-pressure legal training, and produces scores that can guide screening, triage, and program evaluation (Boo et al., 2021; Jia et al., 2022). This measurement gap limits the capacity to identify at-risk students, tailor interventions, and assess the impact of curricular or service innovations.

### 1.4. The Present Study

To address these gaps, the present study develops and validates the FCIS for Chinese undergraduate law students. Guided by OFTP, the FCIS targets three interrelated but distinct facets of insecurity: future career uncertainty, future career self-doubt, and future career anxiety. Our aim is threefold. First, at the measurement level, we seek a brief instrument with a clear dimensional structure, strong internal consistency, and evidence of convergent validity with established indicators of psychological distress. Second, at the service level, we aim to provide a tool that can inform screening, stepped-care triage, and program evaluation within campus health and counseling systems. Third, we seek to establish that the FCIS captures career-specific cognitive appraisals and affective reactions associated with perceived constraints in occupational futures, thereby extending beyond general measures of emotional distress such as anxiety or stress.

We followed a multi-phase process beginning with item generation and expert review. The initial item pool was developed through a comprehensive review of theoretical and empirical literature on job insecurity, career anxiety, and occupational future time perspective (OFTP) (Boo et al., 2021; Carstensen, 2006; Y. Chen et al., 2025a; Gao et al., 2025; Jia et al., 2022; Spurk et al., 2022; Zacher & Frese, 2009). To ensure contextual relevance, this review was supplemented by informal discussions with 20 undergraduate law students and 5 academic advisors regarding common career-related stressors in the legal education context. Based on these theoretical and contextual sources, two authors drafted an initial pool of items emphasizing conceptual clarity, conciseness, and applicability to law-training contexts. Three experts in educational psychology and higher education independently evaluated the items for relevance, clarity, and dimension alignment on a 3-point scale. Items receiving low mean ratings or inconsistent assignment were revised or removed, resulting in a refined 19-item pool for subsequent psychometric testing. This systematic, theory-driven approach enhanced transparency and reproducibility in the item development phase, ensuring conceptual alignment with the OFTP framework.

## 2. Methods

This section describes the development of the FCIS and the procedures used to evaluate its psychometric properties across two independent samples of undergraduate law students in China. We first detail item generation, expert review, and translation and cultural adaptation, followed by participants, procedures, measures, and the statistical analysis plan for Study 1 (exploratory factor analysis) and Study 2 (confirmatory factor analysis and validity testing).

### 2.1. Item Development and Expert Review

Items were generated to capture three theorized facets of future career insecurity in undergraduate legal education: Future Career Anxiety (FCA), Future Career Self-Doubt (FCS), and Future Career Uncertainty (FCU). Two authors drafted an initial pool emphasizing clarity, brevity, and relevance to law-training contexts. Three scholars in educational psychology and higher education independently evaluated each item on 3-point scales for relevance, clarity, and dimension assignment. Items with a mean relevance or clarity below 2.0 or inconsistent assignment were removed or revised. This process eliminated three items and refined seven, yielding a pool of 19 items for psychometric testing (see Appendix A).

### 2.2. Translation

The FCIS items were originally developed in Mandarin Chinese to ensure linguistic and contextual relevance for law students in mainland China. The Chinese version was used for data collection. For publication purposes, all items were subsequently translated into English using a forward–back translation procedure to ensure conceptual and semantic equivalence. Two bilingual translators independently produced forward translations, and discrepancies were reconciled through discussion. A separate bilingual translator then conducted blind back-translation. An expert panel reviewed both versions for semantic and experiential equivalence and approved minor wording adjustments. The original Chinese version of the initial 19-item pool of the FCIS is provided in Appendix A.

### 2.3. Participants and Setting

Study 1. Participants were 447 undergraduate law students from five universities in China: Beijing, Shanghai, Guangzhou, Wuhan, and Xuzhou. Recruitment was conducted through departmental mailing lists and student organizations, and participation was entirely voluntary. The sampling strategy was non-probabilistic and convenience-based, as surveys were distributed to classes and online forums accessible to the research team. Of approximately 600 students invited, 447 provided complete and valid responses, yielding a response rate of roughly 74.5%. The mean age was 20.28 years (SD = 1.22), and 63.09% identified as female. Although convenience sampling may have introduced self-selection bias, the sample reflected a broad distribution across academic years and institutions, enhancing representativeness within the context of Chinese undergraduate law education.

Study 2. An independent sample of 432 undergraduate law students was recruited using the same procedures and inclusion criteria, drawn from additional course cohorts and collaborating universities to ensure non-overlapping participants. Of roughly 600 invitations distributed, 432 complete responses were obtained (response rate ≈ 72.0%). The mean age was 20.72 years (SD = 1.07), with 59.03% female and 40.97% male. As in Study 1, participation was voluntary, anonymous, and uncompensated. While the use of convenience sampling limits generalizability, it was considered appropriate for initial scale validation, as it ensured broad institutional representation across major regions of China.

### 2.4. Procedure and Ethics

Recruitment occurred via official student communication platforms at participating universities. Surveys were completed anonymously online after electronic informed consent. Participation was voluntary, and no incentives were offered. The study protocol received approval from the affiliated institutional review board.

### 2.5. Measures

**Future Career Insecurity Scale (FCIS).** The initial FCIS pool comprised 19 items designed to assess three facets of future career insecurity: FCA, FCS, and FCU. Items were rated on a 5-point Likert scale (1 = “strongly disagree” to 5 = “strongly agree”), with higher scores indicating greater insecurity. Subscale scores are computed as the mean of items loading on each factor; a total FCIS score can be computed as the mean across retained items (reported in Table 1). The final validated instrument retained 13 items (see Table 2), with factor structure confirmed in Study 2.

**Depression Anxiety Stress Scales-21 (DASS-21).** The Chinese version of the DASS-21 (Lovibond & Lovibond, 1995) was used, which consists of 21 items forming three 7-item subscales (Depression, Anxiety, Stress). Participants rated the past week on a 4-point scale (0 = “did not apply to me at all” to 3 = “applied to me very much, or most of the time”). Subscale scores were summed per standard guidance; higher scores reflect greater distress. In Study 2, internal consistencies were α = 0.91 (Depression), α = 0.89 (Anxiety), and α = 0.91 (Stress).

**Sociodemographic Characteristics.** Participants self-reported age (in years), gender, year of study (e.g., first through fourth year), and institution/city of enrollment. No personally identifying information was collected.

### 2.6. Statistical Analysis

**Study 1 (EFA).** We conducted an EFA using principal axis factoring (PAF) to model common variance (Fabrigar et al., 1999), in IBM SPSS Statistics version 29.0. Given expected correlations among factors, we applied Promax (oblique) rotation (Osborne et al., 2008), and a parallel analysis was conducted in R using the Paran package. Factor retention followed Kaiser’s criterion (eigenvalues ≥ 1.00) and scree-plot inspection (Zwick & Velicer, 1986). Items were retained when they exhibited a primary loading ≥ 0.40 on a single factor, no salient cross-loading (≥0.40 on multiple factors), and clear conceptual alignment with the target construct (Howard, 2015).

**Study 2 (CFA and convergent validity).** We tested the correlated three-factor structure (FCA, FCS, FCU) identified in Study 1 using robust maximum likelihood (MLR) in the lavaan package in R (Rosseel, 2012). Model fit was evaluated with *χ*^2^, CFI, TLI, RMSEA (90% CI), and SRMR. Following Hu and Bentler (1999), CFI/TLI ≥ 0.95 and RMSEA ≤ 0.06 indicate good fit, whereas CFI/TLI ≥ 0.90 and RMSEA ≤ 0.08 indicate adequate fit; SRMR ≤ 0.08 was taken as acceptable. We also compared the correlated three-factor model with a unidimensional model (where all 13 items load on a single factor) and a second-order model to evaluate whether FCIS is best represented as one overarching construct or as distinct but correlated dimensions, consistent with the OFTP framework. Model selection was considered based on absolute and incremental fit, as well as practical differences (∆CFI ≥ 0.01; ∆TLI ≥ 0.01; ∆RMSEA ≥ 0.015).

Internal consistency was assessed using Cronbach’s α and McDonald’s ω for each subscale and for the total FCIS score. Convergent validity was examined via two-tailed Pearson correlations between FCIS scores (total and subscales) and DASS-21 subscales (Depression, Anxiety, Stress). We anticipated small-to-moderate positive associations (r ≈ 0.30–0.50), consistent with related but non-isomorphic constructs and the theoretical overlap between affective distress (e.g., anxiety/tension) and career-related insecurity (e.g., anticipatory anxiety, self-doubt) (Lovibond & Lovibond, 1995). Therefore, it provided an appropriate comparative reference for the initial validation of the FCIS.

**Measurement Invariance Across Gender.** To further evaluate the stability of the FCIS factor structure across gender groups, we conducted a series of multi-group confirmatory factor analyses (MG-CFA) to test measurement invariance across gender, implemented in R using the lavaan package (Rosseel, 2012). A hierarchical sequence of models was estimated, including configural, metric, scalar, and residual invariance, following established procedures (F. F. Chen, 2007). Model fit was evaluated using *χ*^2^, CFI, TLI, RMSEA, and SRMR. Changes in fit indices between nested models (∆CFI ≤ 0.010, ∆RMSEA ≤ 0.015, ∆SRMR ≤ 0.010) were used to determine invariance.

## 3. Results

### 3.1. Study 1: EFA

**Figure 1:**
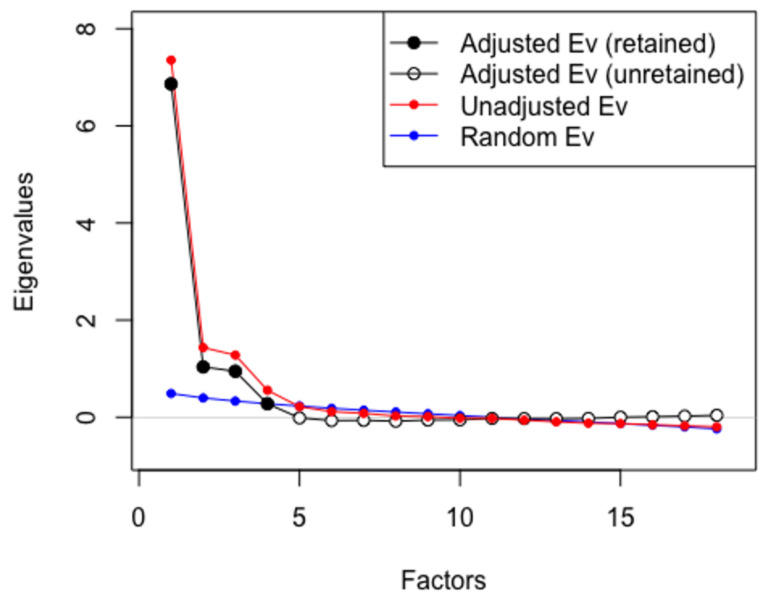
Parallel Analysis Scree Plot.

Sampling adequacy was excellent (KMO = 0.95), and Bartlett’s test of sphericity was significant, *χ*^2^(171) = 7280.26, *p* < 0.001, indicating that the correlation matrix was suitable for factor extraction. The parallel analysis scree plot suggested a three-factor solution (see Figure 1). Using principal axis factoring with Promax rotation, the analysis yielded a clear three-factor solution corresponding to FCA, FCS, and FCU. After two iterative refinement rounds that removed items with suboptimal primary loadings or salient cross-loadings, 13 items were retained. The three extracted factors jointly explained 62.7% of the total variance (Component 1 = 23.3%, Component 2 = 20.8%, Component 3 = 18.6%). After two iterative refinement rounds, 13 items were retained, and five items (FCU3, FCU4, FCS2, FCS5, and FCA6) were excluded due to weak or cross-loadings. Retained items loaded ≥ 0.40 on their intended factor and showed no salient cross-loadings (≥0.40). Rotated pattern loadings are reported in Table 2.

### 3.2. Study 2: CFA, Internal Consistency, and Convergent Validity

**Figure 2:**
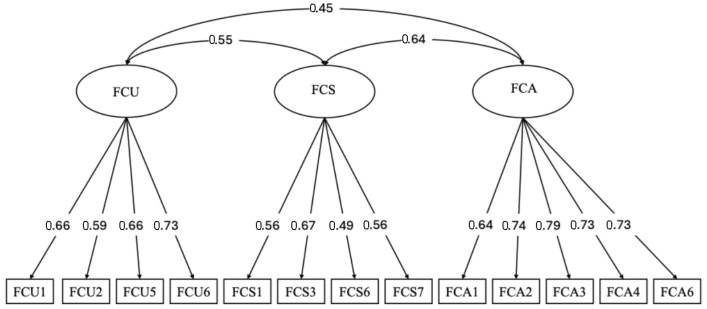
CFA of the FCIS: Standardized Loadings for Three Correlated Factors (Study 2). ***Notes.*** FCU = Future Career Uncertainty; FCS = Future Career Self-Doubt; FCA = Future Career Anxiety. The diagram displays standardized parameter estimates (all *p* < 0.001). Double-headed arrows represent correlations among the latent factors.

A correlated three-factor model specifying FCA, FCS, and FCU was tested via maximum likelihood estimation. Global fit indices indicated acceptable model fit: *χ*^2^(72) = 189.25, *p* < 0.001; CFI = 0.933; TLI = 0.927; RMSEA = 0.061, 90% CI [0.051, 0.072]. All standardized factor loadings were statistically significant and ranged from 0.49 to 0.79 (*p* < 0.001), supporting strong relations between items and their intended latent factors (see Figure 2). For dimensionality, we compared this model with a unidimensional alternative (all 13 items loading on one factor). The one-factor model fit substantially worse, *χ*^2^(65) = 520.28; *χ*^2^/df = 8.00; CFI = 0.740; TLI = 0.688; RMSEA = 0.127; SRMR = 0.097. Differences exceeded conventional thresholds (∆CFI ≥ 0.01; ∆TLI ≥ 0.01; ∆RMSEA ≥ 0.015), supporting a multidimensional structure.

**Figure 3:**
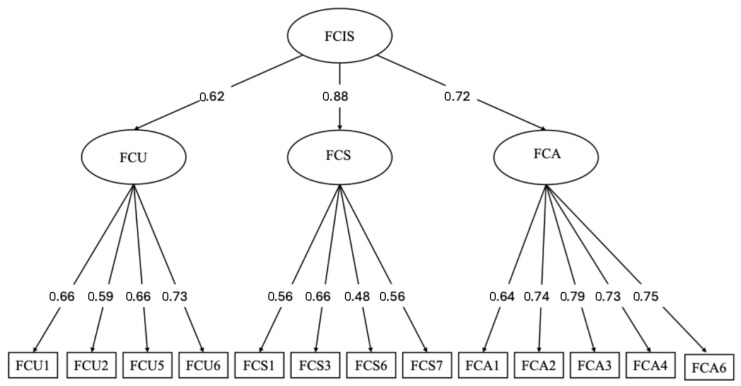
Second-order model. ***Notes.*** FCIS = Future Career Insecurity Scale; FCU = Future Career Uncertainty; FCS = Future Career Self-Doubt; FCA = Future Career Anxiety. The diagram displays standardized parameter estimates, all of which are statistically significant (*p* < 0.001). Double-headed arrows represent correlations among the latent factors.

In addition, a second-order factor model (see Figure 3) was estimated to examine whether the three-factor dimensions reflected a higher-order latent construct representing general future career insecurity. The second-order model indicated acceptable model fit: *χ*^2^(62) = 153.05, *p* < 0.001; CFI = 0.948; TLI = 0.935; RMSEA = 0.058, 90% CI [0.046, 0.071]. These results suggest that while the three dimensions are distinct, they can be meaningfully represented under an overarching higher-order construct of future career insecurity.

Internal consistency was high across subscales and the total scale: FCA (α = 0.83, ω = 0.83), FCS (α = 0.85, ω = 0.85), FCU (α = 0.82, ω = 0.82), and total FCIS (α = 0.87, ω = 0.86), indicating satisfactory internal reliability at both the subscale and overall levels. Convergent validity was supported by significant positive correlations between FCIS scores (total and subscales) and the DASS-21 subscales (Depression, Anxiety, Stress), indicating that higher future career insecurity was associated with greater psychological distress (all *p* < 0.001). As hypothesized, FCA showed the strongest associations with DASS-Anxiety and DASS-Stress, FCS correlated most with DASS-Depression and DASS-Stress, and FCU displayed small-to-moderate correlations across distress domains. Full correlation results are presented in Table 3.

To examine whether the FCIS operates equivalently across gender, multi-group confirmatory factor analyses (MG-CFA) were conducted using the maximum likelihood estimation with robust standard errors (MLR) method in the lavaan package (R version 4.3.2). MLR was selected because it provides robust fit statistics and standard errors under moderate deviations from multivariate normality. Measurement invariance was tested sequentially at the configural, metric, scalar, and residual levels, and invariance was considered supported when changes in fit indices between nested models were within recommended cutoffs (∆CFI ≤ 0.010, ∆TLI ≤ 0.010, ∆RMSEA ≤ 0.015, ∆SRMR ≤ 0.010) (F. F. Chen, 2007). These findings support full measurement invariance across gender.

As shown in Table 4, model fit of the second-order model indices indicated acceptable fit at all levels, and the observed changes in fit indices were within these thresholds, supporting full measurement invariance across gender.

Because scalar invariance was established, latent mean differences were examined with males as the reference group (means fixed to 0). Female students demonstrated significantly higher latent means on all three FCIS dimensions: Future Career Anxiety (ΔM = 0.49, SE = 0.05, z = 10.72, *p* < 0.001), Future Career Self-Doubt (ΔM = 0.20, SE = 0.02, z = 9.26, *p* < 0.001), and Future Career Uncertainty (ΔM = 0.40, SE = 0.03, z = 12.28, *p* < 0.001). These results suggest that female students experienced greater overall career-related insecurity than their male counterparts.

## 4. Discussion

Across two independent samples of Chinese undergraduate law students, we developed and validated the FCIS as a concise, multidimensional assessment of future-oriented career distress. FCIS showed a stable three-factor structure (FCA, FCS, and FCU) with good overall model fit and high internal consistency. All factor loadings were statistically significant, and FCIS scores correlated positively with depression, anxiety, and stress, supporting convergent validity (see Figure 2 and Table 3). Taken together, these results indicate that FCIS is both psychometrically robust and practically feasible for use in legal education settings.

### 4.1. Theoretical Implications

Findings give the OFTP framework greater specificity in undergraduate legal training (Y. Chen et al., 2025a; Gao et al., 2025; Zacher & Frese, 2009). When students perceive limited time and opportunity, a constrained future perspective appears to manifest through separable but correlated channels: cognitive appraisals about predictability of pathways and outcomes (FCU) and about capability/attainability (FCS), and an affective response reflecting anticipatory arousal (FCA). The replicated three-factor solution and its superior fit over a unidimensional alternative support the view that future career insecurity is not a single, undifferentiated state, but rather a constellation of appraisals and affect that co-occur under perceived constraint (F. F. Chen, 2007; Spurk et al., 2022).

The convergent validity pattern reinforces this interpretation. FCA related most strongly to anxiety and stress, consistent with threat appraisal and heightened arousal; FCS related more to depressive affect and stress, consistent with reduced perceived capability and controllability; FCU showed small-to-moderate links across distress domains, consistent with ambiguity acting as a cognitive driver that can potentiate both worry and demoralization when routes into practice are unclear (Chan et al., 2024; Lazarus, 1984). Together, the evidence suggests a sequential process for future tests: perceived constraints on time/opportunity narrow OFTP, increasing uncertainty about pathways (FCU), and lowering perceived capability (FCS), which in turn elevate anxiety (FCA) (Y. Chen et al., 2025a; Gao et al., 2025; Zacher & Frese, 2009).

### 4.2. Positioning Relative to Existing Measures

FCIS complements but is not redundant with prevalent instruments. Job-insecurity measures were developed for employed workers and emphasize the threat of job loss or contract instability rather than student transitions into work (De Witte et al., 2016). Impostor-phenomenon scales capture global self-evaluation and fear of exposure as inadequate, but they do not index future-career structure or sustained anticipatory arousal specific to entry into legal practice (Clance & Imes, 1978; Skead & Rogers, 2014). General indicators of career anxiety or work-life uncertainty often operationalize a single affective dimension or use domain-general items, which limits the diagnostic precision for targeted campus interventions (Boo et al., 2021; Jia et al., 2022; Spurk et al., 2022).

By contrast, the FCIS is brief, student-specific, and theory-aligned: differentiating between uncertainty about pathways (FCU), self-doubt about capability (FCS), and anxiety (FCA) in a high-stakes professional training context. Although the FCA dimension shares affective characteristics with general anxiety, the FCIS captures domain-specific cognitions and emotions that are uniquely tied to students’ perceptions of their occupational futures. The positive but moderate correlations between FCIS subscales and the DASS-21 dimensions of anxiety and stress indicate related but non-redundant constructs, consistent with evidence that domain-specific future concerns (e.g., occupational or academic) constitute situated expressions of broader affective dispositions (Boo et al., 2021; Gao et al., 2025; Zacher & Frese, 2009). Furthermore, the three-factor structure that differentiates uncertainty, self-doubt, and anxiety supports a multidimensional conceptualization of career insecurity rather than a single, generalized distress factor. This pattern aligns with OFTP-based models, suggesting that future-oriented distress involves separable cognitive (uncertainty, self-doubt) and affective (anxiety) pathways arising from perceived temporal and opportunity constraints (F. F. Chen, 2007; Y. Chen et al., 2025a; Gao et al., 2025). Thus, the FCIS extends beyond general anxiety measures by delineating the specific mechanisms through which occupational future construal influences students’ psychological well-being. This dimensional profile is actionable for screening, stepped-care triage, and program evaluation in university settings, while remaining compact enough for repeated administration during pressure points in the academic calendar (Antonio & Chiesa, 2024; Zacher & Frese, 2009).

### 4.3. Practical Implications

This work extends the OFTP framework to the university-to-work transition and provides a brief tool that differentiates FCU, FCS, and FCA in future-career distress among law students (Zacher & Frese, 2009). The FCIS can be used to identify students at elevated risk of career-related stress, disengagement, or broader psychological distress and to align support accordingly.

Observed differences in latent means reflect genuine gender differences in future-career insecurity rather than measurement bias or scale non-equivalence. In practical terms, it confirms that the FCIS can be used to compare the average levels of future-career anxiety, self-doubt, and uncertainty between men and women with confidence that such comparisons are fair and unbiased.

Attaining residual invariance further strengthens this conclusion by demonstrating that the item-specific residual variances are equivalent across groups, indicating that the amount of unexplained measurement error is consistent for both men and women. This suggests that the FCIS functions with comparable precision across gender, enhancing its suitability for both individual assessment and group-level monitoring. Together, these results validate the use of latent mean comparisons reported above and reinforce the FCIS’s applicability in identifying gender-specific patterns of future-career distress without confounding measurement artifacts.

Beyond general career-insecurity measures, the FCIS makes three contributions. First, it captures discipline-specific stressors that are salient in legal education, such as competitive ranking, professional gatekeeping, and normative expectations of prestige that are not represented in general instruments developed for employed adults (De Witte et al., 2016; Spurk et al., 2022). Second, it incorporates culturally grounded features relevant to Chinese students, including Confucian values surrounding family honor and occupational prestige (Ng & Feldman, 2007; Zhu et al., 2021). Third, its multidimensional structure distinguishes between cognitive appraisals (uncertainty and self-doubt) and affective reactions (anxiety), providing theoretically coherent subscales that can guide both individual interventions and institutional policy.

At the student level, subscale profiles can guide supports: elevated FCU suggests pathway clarification, labor-market information, and structured exposure to professional tasks; elevated FCS points to mentored practice, skills feedback, and cognitive reframing to strengthen capability beliefs; elevated FCA indicates brief anxiety-management and intolerance-of-uncertainty strategies in counseling or coaching (Alnajjar & Abou Hashish, 2024). Brief FCIS screening can be scheduled at predictable high-stress points (e.g., internship placement, on-campus interviews, bar-exam preparation) to triage students to proportionate care.

At the program level, aggregated FCIS data can flag system issues (e.g., opaque recruitment routes, misaligned expectations) and inform curriculum adjustments and resource allocation. Because the instrument is brief, it is feasible to embed in existing advising, career-services workshops, and counseling intake without adding burden. Using repeated administrations, units can also evaluate the impact of curricular or service innovations by tracking changes in subscales over time (Antonio & Chiesa, 2024; Y. Chen et al., 2025b).

### 4.4. Limitations and Future Directions

Despite the contributions of this study, several limitations should be acknowledged. First, the sample consisted of full-time undergraduate law students in China, recruited through convenience sampling, which may limit the representativeness and generalizability of the findings. Future research should employ probability or stratified sampling methods and examine whether the FCIS structure and correlates hold across other disciplines, professions, and cultural contexts through multi-group invariance testing.

Second, the present study was cross-sectional, precluding inferences about temporal stability. Given that the FCIS is intended as a brief and relatively stable measure of future career insecurity, future studies should conduct test–retest reliability analyses to establish short-term stability and assess whether the instrument captures enduring rather than transient affective and cognitive states.

Third, although the DASS-21 was used as an external criterion to evaluate convergent validity, it primarily measures general psychological distress and not vocationally specific concerns. The observed correlations should therefore be interpreted cautiously. Future validation work should incorporate construct-relevant measures, such as the Career Anxiety Scale, Career Adapt-Abilities Scale, or Perceived Employability Inventory, to better contextualize the FCIS within the vocational domain.

Finally, regarding conceptual novelty, the FCIS extends existing professional anxiety scales by targeting the university-to-work transition and by distinguishing cognitive (uncertainty and self-doubt) and affective (anxiety) facets of career insecurity within a culturally grounded and discipline-specific context. Continued research should examine the predictive and discriminant validity of the FCIS in relation to existing occupational anxiety instruments to further substantiate its theoretical contributions.

## 5. Conclusions

The present study developed and validated the FCIS as a multidimensional measure of career-related psychological distress among Chinese law students. By empirically distinguishing between future career uncertainty, self-doubt, and anxiety, the FCIS offers a theoretically informed and psychometrically robust tool for assessing students’ cognitive and emotional responses to anticipated career difficulties. The scale offers a novel measurement for research and has practical utility for early identification, intervention, and policy efforts to support students’ occupational well-being.

## Figures and Tables

**Table 1:** Mean, SD, Skewness and Kurtosis of the initial 19 items.

	Mean	*SD*	Skewness	Kurtosis
**Future Career Uncertainty (FCU)**				
FCU1.	3.23	0.82	0.468	0.548
FCU2.	3.33	0.99	0.703	0.531
FCU3.	3.30	0.86	0.554	0.546
FCU4.	3.29	0.88	0.650	0.851
FCU5.	3.76	0.99	0.374	−0.085
FCU6.	3.59	0.93	0.444	0.197
**Future Career Self-doubt (FCS)**				
FCS1.	3.22	0.85	0.733	1.134
FCS2.	3.32	0.95	0.755	0.703
FCS3.	3.26	0.79	0.604	1.054
FCS4.	3.75	0.93	0.412	0.213
FCS5.	3.69	0.92	0.277	0.200
FCS6.	3.48	0.85	0.577	0.797
FCS7.	3.43	0.98	0.320	−0.0227
**Future Career Anxiety (FCA)**				
FCA1.	3.49	1.01	0.252	−0.423
FCA2.	3.57	1.04	0.144	−0.551
FCA3.	3.42	1.03	0.228	−0.582
FCA4.	3.12	0.95	0.683	0.226
FCA5.	3.49	1.03	0.268	−0.406
FCA6.	3.36	1.05	0.373	−0.525

**Table 2:** EFA Pattern Loadings for the Final 13 FCIS Items (Study 1).

Item	Factor 1: FCA	Factor 2: FCS	Factor 3: FCU
FCA2. I feel tense when I think about meeting the expectations of future employers, colleagues, or clients. 当想到需要满足未来雇主、同事或客户的期望时，我会感到紧张。	**0.851**	0.275	0.247
FCA1. I feel anxious about securing stable or desirable employment in the legal field. 我因能否在法律领域获得稳定或理想的工作而感到焦虑。	**0.811**	0.201	0.208
FCA3. I worry about the financial stability of a legal career. 我担心法律职业的经济稳定性。	**0.78**	0.27	0.286
FCA4. I feel overwhelmed when I think about the workload and pressure in legal practice. 一想到法律实务的工作量与压力，我就感到不堪重负。	**0.808**	0.21	0.264
FCA5. I feel uneasy about whether a legal career will be personally meaningful for me. 我对法律职业是否能让我获得个人意义感到不安。	**0.647**	0.201	0.305
FCS7. I question whether pursuing law was the right decision for my long-term career. 我质疑攻读法律是否是我长远职业发展的正确选择	0.205	**0.782**	0.198
FCS3. I question whether my current professional connections are sufficient for career advancement. 我质疑自己当前的人脉与职业资源是否足以支持职业晋升。	0.168	**0.828**	0.226
FCS6. I doubt my contributions in legal discussions or professional settings will be taken seriously. 我质疑自己在法律讨论或职业场合中的观点能否得到应有的重视	0.332	**0.57**	0.314
FCS1. I doubt that my legal reasoning and argumentation are strong enough for professional practice. 我怀疑自己的法律推理与论证能力是否足以胜任专业实践。	0.338	**0.531**	0.318
FCU2. I am unsure how my current academic progress will map onto hiring routes in legal practice. 我不清楚当前的学业进展将如何对应法律实务的入职渠道。	0.216	0.241	**0.871**
FCU6. I am uncertain whether the effort I invest now will lead to meaningful opportunities in the legal field. 我不确定自己现在投入的努力是否会带来在法律领域有意义的机会。	0.297	0.264	**0.698**
FCU1. I am uncertain whether my academic achievements (e.g., grades, recognition, and scholarships) accurately signal my abilities to potential employers in law. 我不确定我的学业成就（如成绩、荣誉和奖学金）能否准确向潜在的法律雇主传达我的能力。	0.248	0.193	**0.518**
FCU5. I am unsure about my next steps because my law program has provided fewer career opportunities than I expected. 由于法学专业/法学院学习提供的职业机会少于我的预期，我对下一步该如何规划不确定。	0.33	0.321	**0.474**

***Notes.*** Extraction = principal axis factoring; rotation = Promax; items were retained when the primary loading was ≥0.40 with no cross-loading ≥ 0.40. Bold values represent factor loadings > 0.40. Total variance explained = 62.7%. *N* = 447. FCA = Future Career Anxiety; FCS = Future Career Self-Doubt; FCU = Future Career Uncertainty; FCU3, FCU4, FCS2, FCS5, and FCA 6 were excluded during the EFA process.

**Table 3:** Correlations Between FCIS Scores and DASS-21 Subscales (Study 2).

Measure	1	2	3	4	5	6	7
**1. FCIS**	–						
**2. FCA**	0.742 ***	–					
**3. FCS**	0.717 ***	0.695 ***	–				
**4. FCU**	0.655 ***	0.697 ***	0.793 ***	–			
**5. Depression**	0.312 ***	0.269 ***	0.364 ***	0.302 ***	–		
**6. Anxiety**	0.573 ***	0.559 ***	0.745 ***	0.551 ***	0.677 ***	–	
**7. Stress**	0.697 ***	0.898 ***	0.636 ***	0.672 ***	0.247 ***	0.491 ***	–

***Notes.*** Pearson correlations (two-tailed). FCIS = total Future Career Insecurity Scale; FCA = Future Career Anxiety; FCS = Future Career Self-Doubt; FCU = Future Career Uncertainty; Depression, Anxiety, Stress = Subscales of DASS-21. *** *p* < 0.001.

**Table 4:** Gender Invariance Results.

Models	*χ* ^2^	df	CFI	TLI	RMSEA	SRMR	ΔCFI	ΔRMSEA	ΔSRMR
M1	213.700 ***	124	0.949	0.935	0.058	0.050			
M2	228.949 ***	144	0.951	0.947	0.052	0.054	0.002	0.012	0.004
M3	245.380 ***	157	0.949	0.950	0.051	0.055	−0.002	0.003	0.001
M4	263.803 ***	166	0.944	0.947	0.052	0.052	−0.005	−0.003	−0.003

***Notes.*** M1 = configural invariance; M2 = metric invariance; M3 = scalar invariance; M4 = residual invariance; CFI = comparative fit index; TLI = Tucker–Lewis index; RMSEA = root mean squared error of approximation; SRMR = standardized root mean squared residual. *** *p* < 0.001.

## Data Availability

The data that support the findings of this study are available from the corresponding author upon reasonable request.

## Abbreviations

The following abbreviations are used in this manuscript:

EFA	Exploratory factor analysis
CFA	Confirmatory Factor Analysis
FCIS	Future Career Insecurity Scale
FCA	Future Career Anxiety
FCU	Future Career Uncertainty
FCS	Future Career Self-Doubt
DASS-21	Depression Anxiety and Stress Scales
OFTP	Occupational Future Time Perspective

## Appendix A. Initial 19-Item Pool of the FCIS After Expert Review

**Dimensions and Items**	
**Future Career Uncertainty (FCU)**	
FCU1.	I am uncertain whether my academic achievements (e.g., grades, recognition, and scholarships) accurately signal my abilities to potential employers in law. 我不确定我的学业成就（如成绩、荣誉和奖学金）能否准确向潜在的法律雇主传达我的能力。
FCU2.	I am unsure how my current academic progress will map onto hiring routes in legal practice. 我不清楚当前的学业进展将如何对应法律实务的入职渠道。
FCU3.	I am unclear about my career prospects in law given what my education currently offers. 基于我目前的法学专业学习，我对自己在法律领域的职业前景不够明确。
FCU4.	I am uncertain whether my available financial resources will allow me to pursue the opportunities needed for a successful legal career. 我不确定现有的经济资源是否足以支持我争取迈向成功法律职业所需的机会。
FCU5.	I am unsure about my next steps because my law program has provided fewer career opportunities than I expected. 由于法学专业/法学院学习提供的职业机会少于我的预期，我对下一步该如何规划不确定。
FCU6.	I am uncertain whether the effort I invest now will lead to meaningful opportunities in the legal field. 我不确定自己现在投入的努力是否会带来在法律领域有意义的机会。
**Future Career Self-doubt (FCS)**	
FCS1.	I doubt that my legal reasoning and argumentation are strong enough for professional practice. 我怀疑自己的法律推理与论证能力是否足以胜任专业实践。
FCS2.	I question my competence to handle the responsibilities expected of a junior lawyer. 我质疑自己是否具备承担初级律师应尽职责的能力。
FCS3.	I question whether my current professional connections are sufficient for career advancement. 我质疑自己当前的人脉与职业资源是否足以支持职业晋升。
FCS4.	I am not confident that my coursework and training were rigorous enough to prepare me for real-world legal work. 我不自信自己的课程与训练是否足够严谨，能够让我为真实世界的法律工作做好准备。
FCS5.	I doubt that my law program has prepared me to work confidently and independently in legal practice. 我怀疑法学专业/法学院学习是否使我具备在法律实务中自信且独立工作的能力。
FCS6.	I doubt my contributions in legal discussions or professional settings will be taken seriously. 我质疑自己在法律讨论或职业场合中的观点能否得到应有的重视。
FCS7.	I question whether pursuing law was the right decision for my long-term career. 我质疑攻读法律是否是我长远职业发展的正确选择。
**Future Career Anxiety (FCA)**	
FCA1.	I feel anxious about securing stable or desirable employment in the legal field. 我因能否在法律领域获得稳定或理想的工作而感到焦虑。
FCA2.	I feel tense when I think about meeting the expectations of future employers, colleagues, or clients. 当想到需要满足未来雇主、同事或客户的期望时，我会感到紧张。
FCA3.	I worry about the financial stability of a legal career. 我担心法律职业的经济稳定性。
FCA4.	I feel overwhelmed when I think about the workload and pressure in legal practice. 一想到法律实务的工作量与压力，我就感到不堪重负。
FCA5.	I feel uneasy about whether a legal career will be personally meaningful for me. 我对法律职业是否能让我获得个人意义感到不安。
FCA6.	I feel anxious that my law program has not prepared me well enough for professional success. 我因觉得法学专业/法学院学习没有让我为职业成功做好充分准备而感到焦虑。
